# On the Treatment of Hysteria

**Published:** 1854-08

**Authors:** 


### *On the Treatment of Hysteria.* By M. Gendrin.

The observation of hysterical disease teaches us that by placing patients in appropriate hygienic conditions and leaving the malady to itself, it will disappear spontaneously as age advances; so that an expectant treatment will ultimately result in recovery. The physician who expects a speedy cure will be disappointed by a disease essentially chronic in its nature and progress.

The practitioner will sometimes be *forced* to adopt the expectant plan, and he should then carefully remove everything which can exasperate the disease, especially such causes as excite the nervous system. If the hysterical woman is unhappy in her domestic relations ; if she is surrounded by those who, not understanding her character, expose her to frequent vexations, she should travel, she should be separated from all that can cause disturbances in her organization ; this is the main hygienic treatment. With moral and physical repose ; a life rendered happy by all kinds of enjoyments, the disease will finish of itself, by passing rapidly through all its phases.

There are, however, methods of treatment and even pharmaceutical preparations which modify the nervous system favorably, and cure hysteria. Authors who have discussed these, have dwelt too much upon remedies appropriate in the paroxysms of the disease. An immense number of remedies have been lauded one after the other, and all of them are sufficiently inert; it is evident that the affection itself is not arrested by these means.

Nevertheless, the treatment of prolonged paroxysms of hysteria is not to be neglected. It consists chiefly in the employment of means which may influence the inertia of the nervous system. By plunging the patient suddenly into cold water, or by employing extensively cold effusion, we may procure a suspension of the clonic spasms, and a cessation of the attack; but if this plan is practiced timidly, it will not be a sufficient sedative, and far from calming, it will exasperate the symptoms. If a glass of water is dashed in the face of a woman laboring under an attack of hysteria, as is so often done, it may indeed cause a termination of the attack in some cases, but in the majority it will exaspirate all the symptoms. It is necessary, in order to obtain a result, to paralyse the nervous system by the rapid abstraction of caloric.

External topical remedies are quite ineffectual; the use of sinapisms, for example, especially in the ecstatic form, constitutes an old woman's treatment which is perfectly useless. They will tell you—"She was so ill that she perceived nothing, and sinapisms were applied so long that they occasioned eschars;" this is not in the least astonishing, when you recollect to what extent anæsthesia exists in these subjects, even in regard to odors.

There are some however, susceptible of the impressions of those fœtid substances, which frequently occasion vomiting and lypothymia even in healthy individuals. In those hysterical patients who preserve the sensibility of the pituitary membrane, we employ these fœted odors to hasten the termination of their attacks; the odors of assafœtida, tincture of castoreum, ether, acetic acid and ammonia, burnt horn and feathers, seem to exercise a happy influence over hysterical attacks, and to favor their cessation.

In many cases the paroxysm is arrested by discovering a portion of the body in which insensibility does not exist, through which the internal organs may be stimulated. If the stomach is swollen, as occurs when the patient has swallowed large quantities of air, the attack may be cut short by thoroughly compressing this viscus. The majority of hysterical women preserve the senibility of vision. During the paroxysms the eye-lids are convulsively closed; the light incommodes the patient. If the lids be forcibly separated, and a bright ray of light be directed upon the eye, the retina will sometimes be so excited as to cause a termination of the attack, At other times this will cause pain and will augment the violence of the spasms. It is a plan worth trying. The same remedies give the same results in the ecstatic form. It is requisite, however, to study the action of these agents on different patients. I have spoken of a lady who recovered from her attacks with the rapidity of the electric spark, under the influence of the feeblest odors; in others some particular odorous substance is required, acetic acid, for example, and with others all may fail.

Some women in hysteria are susceptible of the liveliest emotions upon hearing certain musical notes, upon listening to an air which they admire from some cause or another, while other tunes make no impression upon them. They are aroused by certain voices and not by others; we should therefore avail ourselves of these special means, to induce the termination of their attacks.

It is therefore necessary to investigate carefully the modes by which the nervous system may be rendered susceptible of impressions, in treating the attacks of clonic and ecstatic hysteria. But this treatment of the attacks does not affect the disease itself; it is an indirect medication only; we must search for therapeutical agents which will influence the pathological state of the nervous system; we must look for curative agents.

Remedies exist which diminish nervous irritability, and interrupt the abnormal manner in which the nervous functions are performed in this disease. Opium, mentioned by Galen as a powerful agent in lessening the aptitude to convulsions, deadens the sensibility of organs whose sensitive functions are normally performed. It loses its narcotic virtues in those subjects whose nervous functions are abnormal. These subjects often acquire the faculty of undergoing the narcotic influence of opium without stupefaction, or else, if hyperæsthesia of the digestive organs exists, which is often the case, it causes vomiting, even in the smallest doses. Opium, therefore, either exagerates the sensibility of the digestive canal, in hysterical persons, or else it is readily supported and its influence is lost, unless it is given in very large doses. Intolerance of this remedy, however, is only observed in a few patients, while insusceptibility to its effects is observed in the majority.

Hysteria is not the only disease in which opium may be given without producing a stupefying effect. Thus, in tetanus, excessive doses of this drug may be administered without the slightest result, or else immediate effects upon the digestive canal are produced, without secondary effects, that is without narcotism.

One hysterical person experiences the immediate and exaggerated effect of opium; another vomits at the dose of a quarter of a grain; and we see others again on whom large doses produce no effect; hence the necessity, when we undertake to destroy the mobility of the nervous system, to proceed by degrees towards that point where the patient is sensible to the therapeutic influence of the medicine, and to stop short of its exaggerated action. Thus, in such a case it will be necessary to administer the opium *gradatim* up to the point where the system feels its influence. One or two grains may be commenced with, and the dose gradually increased. In the case of the woman occupying No. 2 *Salle de Rosaire*, there was an exacerbation of the disease every time she was changed to a different ward, and we gave 34 grains of opium without obtaining any obvious signs of narcotism; nevertheless she was considerably nauseated, proving that the medicine acted directly upon the stomach, and I have already reminded you that it was at this point that sensibility returned to the skin of the face. With some patients, it is not necessary to go so far to obtain the same result. Thus, in the case of the woman occupying No. 23 in the same ward, we observed the narcotic effects after six grains had been given. But in this case, we were obliged to stop the medicine on account of manifest symptoms of typhus.

In the case of hysterical patients who bear opium badly even in the most feeble doses, incorrigible vomitings and violent cardialgic pains are induced, no matter in what manner it be taken internally, or even though it be applied endermically. I have seen a quarter of a grain of opium applied upon a vesication induce vomiting. With these patients, narcotism cannot be induced, and the medicine is not applicable; but in most cases the opium acts by properly varying the dose, and the sedative effect may be observed as soon as nausea begins.

Experience will teach you that by this modification of the treatment, which ought in most cases, be continued a long time, with many hysterical patients you will lessen by degrees the exaggerated mobility of the nervous system, reëstablish the equilibrium between the perception of sensitive impressions and the cause producing them; and when the sensibility is once reëstablished, the attacks will disappear; often indeed, they disappear before the restoration of the sensibility. This is the conception we have of the effect of narcotics in relieving the nervous system of abnormal irritability, and of their utility in this affection.

In cases where opium is badly borne, it may be made more tolerable by combination with ether, which has the effect of combatting the narcotism caused by the medicine. Ether is a diffusible stimulant, but sedative upon the nervous system, and acts by calming the convulsive movements which throw the patients into a sort of torpor. It also excites the circulation and intellectual faculties. We believe that the ether will only act in large doses. It is generally given, like musk, in feeble doses, say from 10 to 40 drops, which is equivalent to not giving it at all. The volatility of the ether is favored by the warmth of the apartment and the evaporation which takes place in the mouth and stomach while it is being swallowed, so that the small amount which reaches the stomach is entirely inert. But in giving this medicine in large doses, you have a marked sedation, not only in hysteria, but in patients whose nervous systems are easily moved. We never give less than two drachms and a half at first, and sometimes carry it to four or five drachms. It must be given in a large quantity of cold water, for, not being soluble in water, its volatilization is avoided, and more marked effects obtained. Its action as a diffusible stimulant is transient, is soon dissipated by the respiratory organs and the effect ceases. To obtain a real effect from the ether, it must be given (in large quantities of water) from ten to twenty times a day.

We have alluded to an exaggerated sensibility, a sort of hyperæsthesia of the digestive organs in hysterical subjects, giving rise to cardialgic pains and cramps of the stomach after the ingestion of food or of medicinal substances. It will be necessary to remove this exaggerated susceptibility of the digestive organs in order to render them sufficiently tolerable of medicines so as to effect a cure. The use of the subnitrate of bismuth furnishes this means, which has the effect of allaying this irritability, and may be given as an adjuvant; thus obviating the pains which of themselves frequently cause a convulsive attack. But, to obtain these results, it is necessary to give this medicine in certain quantities. Many, and especially the disciples of Reil, recommend it in very large doses. Generally, it is sufficient to give from twenty to thirty grains, in which dose it exercises an incontestable sedative effect, diminishing the irritability of the stomach, and permitting the patient to retain the opium and the food. The pain complained of in the digestive organs modifies the gastric and hepatic secretions, and frequently instead of cardialgic pains we have a veritable dyspepsia. The patient suffers from sour eructations, acrid deposits around the teeth, constipation, inability to take acid substances, etc., etc.; leaving the stomach in such a condition that it can neither bear food or medicine; the derangement producing such an influence upon the nervous system that it is frequently the cause of hysterical attacks. The alkaline remedies, to some extent, relieve these conditions.

We have not spoken of all the medicines by which, in stupefying the nervous system, hysterical attacks may be prevented. Opium and ether only have been alluded to, but there are others which may be used, though required in very large quantities. Thus, castoreum has been favorably regarded by many; but, like the ether, to produce any effect, it must be given, not in the small doses usually spoken of, which are useless, but to the amount of thirty or forty grains. We prefer it in substance to tincture, as all of its properties are not soluble in alcohol.

Assafœtida has been recommended for the same purpose. Apart from the sedative action of its fœtid odor, and its purgative effect, we believe it has little power. Being very insoluble, when given in the form of pill, there is great danger of its passing off by the bowels, without being dissolved. Used in the form of injection, it acts as a purgative, but its anti-spasmodic action is not very evident. It is useless to pass in review all the medicines recommended in hysteria. All the stupefants, in acting directly upon the nervous system, may lessen the manifestations and cure hysteria gradually: thus orange leaves, valerian, linden, etc., etc., which act upon the nervous system in a direct manner by means of their odorous principle, but which never effect, like opium, a direct cure.

We are obliged to resort to empiricism in order to explain the action of these remedies, for it is only experience that teaches us to appreciate their effects. Experience teaches us that opium is a powerful anti-spasmodic; it acts in a preëminent degree upon the intellectual faculties, and has no influence upon the organs of sense like the odoriferous substances, to incommode patients and provoke hysterical attacks. But while there is intestinal anæsthesia, there is no action on the part of opium: it may then be given in large doses without producing any effect upon the digestive tube or upon the circulation, and in such cases, notwithstanding its powerful anti-spasmodic attributes, it has no influence over hysteria.

To pretend to cure hysteria by medicine without regulating the regimen is futile. It is necessary to inquire into the irregular habits of patients and remedy them; to regulate their sleep, and adjust their occupations properly, for often they turn day into night, and vice versa. There are subjects who use themselves to excitement of the organs of sense, such as inhaling strong odors, inordinate passion for music, living in the midst of high excitation, etc., etc. All these habits retard a cure by the excitement which they keep up in the nervous system. Masturbation, which is very common with hysterical subjects, prevents a cure. It is the same with light garments—some pinch their feet like the Chinese, while others delight in tight corsets. These are vicious habits, as they modify the nervous system and protract the cure. Those who are too sedentary must be made to take exercise in the open air, as a curative means. In directing the nervous influence upon the locomotion the perception of sensitive impressions is prevented, thus avoiding superexcitation of the nervous system, which brings on attacks of hysteria.

In conclusion, a regular hygiene, avoiding the influence of external stimulants, the use of sedatives applied to the periphery of the body, by means of the cold bath conveniently prolonged, constitutes the best medication for hysteria. Add to this opium in large doses, adjusted according to the susceptibility of the patients; subnitrate of bismuth to regulate the irritability of the stomach, and cause it to support the necessary medicine and nourishment; the proper absorbent alkalies for dyspeptic conditions: and the martial preparations when there is chlorosis.

In applying this medication to hysteria a number of cases may be frequently cured. Nevertheless, it is a disease essentially chronic, as we have said, and will often require a chronic treatment. One, two, or sometimes more years may be neceasary, but by perseverence an amelioration will certainly be effected. Such is the result obtained unless there should be complication with epilepsy; then the prognosis is very different.—*Gazette des Hôpitaux*, from *Virginia Med. and Surg. Journal.*

